# Assessment of the potential health risks associated with the aluminium, arsenic, cadmium and lead content in selected fruits and vegetables grown in Jamaica

**DOI:** 10.1016/j.toxrep.2017.03.006

**Published:** 2017-03-29

**Authors:** Johann M.R. Antoine, Leslie A. Hoo Fung, Charles N. Grant

**Affiliations:** International Centre for Environmental and Nuclear Sciences, 2 Anguilla Close, University of the West Indies, Mona Campus, Kingston 7, Jamaica

**Keywords:** AAS, atomic absorption spectrophotometry, ATSDR, Agency for Toxic Substances & Disease Registry, EDI, estimated daily intake, FAO, Food and Agriculture Organization of the United Nations, GTHQ, global target hazard quotient, HI, hazard index, IAEA, International Atomic Energy Agency, INAA, instrumental neutron activation analysis, JECFA, Joint FAO/WHO Expert Committee on food additives, LOAEL, lowest observed adverse effect level, NATA, National Air Toxics Assessment, NIST, National Institute of Standards and Technology, NOAEL, no observed adverse effect level, PTWI, provisional tolerable weekly intake, R*f*D, oral reference dose, RSL, regional screening levels, TCR, target cancer risk, THQ, target hazard quotient, US EPA, United States Environmental Protection Agency, WHO, World Health Organization, Risk assessment, Heavy metals, Target hazard quotient, Target cancer risk, Hazard index, Food, Jamaican crops, Estimated daily intake

## Abstract

Thirteen Jamaican-grown food crops − ackee (*Blighia sapida*), banana (*Musa acuminate*), cabbage (*Brassica oleracea*), carrot (*Daucus carota*), cassava (*Manihot esculenta*), coco (*Xanthosoma sagittifolium*), dasheen (*Colocasia esculenta*), Irish potato (*Solanum tuberosum*), pumpkin (*Cucurbita pepo*), sweet pepper (*Capsicum annuum*), sweet potato (*Ipomoea batatas*), tomato (*Solanum lycopersicum*) and turnip (*Brassica rapa*) − were analysed for aluminium, arsenic, cadmium and lead by atomic absorption spectrophotometry and instrumental neutron activation analysis. The fresh weight mean concentrations in these food crops (4.25–93.12 mg/kg for aluminium; 0.001–0.104 mg/kg for arsenic; 0.015–0.420 mg/kg for cadmium; 0.003–0.100 mg/kg for lead) were used to calculate the estimated daily intake (EDI), target hazard quotient (THQ), hazard index (HI) and target cancer risk (TCR) for arsenic, associated with dietary exposure to these potentially toxic elements. Each food type had a THQ and HI < 1 indicating no undue non-carcinogenic risk from exposure to a single or multiple potentially toxic elements from the same food. The TCR for arsenic in these foods were all below 1 × 10^−4^, the upper limit used for acceptable cancer risk. There is no significant health risk to the consumer associated with the consumption of these Jamaican-grown food crops.

## Highlights

• Thirteen food crops were analysed for aluminium, arsenic, cadmium and lead.
• Mean concentrations were used to calculate EDI, THQ and HI.
• TCR was calculated for arsenic for all food crops.
• The THQ and HI were <1 for all food crops; target cancer risk did not exceed 10^−4^.
• The food crops evaluated pose no undue risk to the consumer.

## 1 Introduction

The primary method of exposure to trace elements from the non-occupationally exposed population is through diet. In the case of nutrition, iron deficiency is considered the most prevalent nutritional deficiency [1]. Inadequate zinc intake is also prevalent as well; it has been estimated that 17.3% of the global population is at risk of zinc deficiency [2]. From a food safety standpoint, the intakes of several trace elements are strictly regulated by several international bodies including the Codex Alimentarius and the Joint FAO/WHO Expert Committee on Food Additives (JECFA), as well as numerous regional and national bodies. In the year 2011, JECFA withdrew the provisional tolerable weekly intake (PTWI) for both lead and inorganic arsenic with the recommendation that the previously established PTWIs could no longer be considered health protective [3], [4]. JECFA has since not re-established a PTWI for either element.

**Fig. 1:**
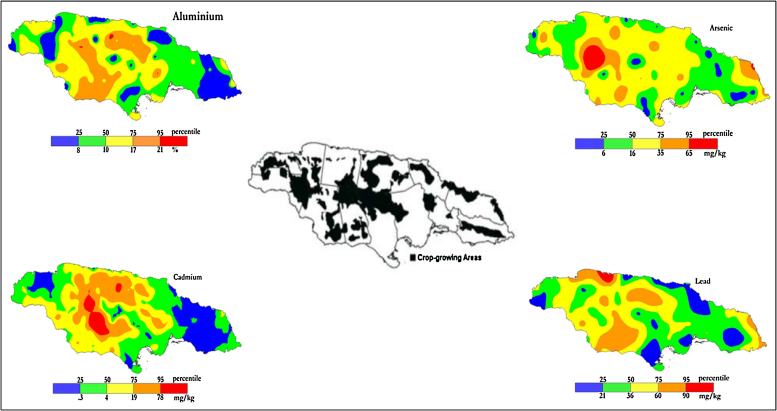
The relationship between small farmer crop-growing areas and the distribution of aluminium, arsenic, cadmium and lead in Jamaican soils.

Geochemical investigations of Jamaican soils have revealed the enrichment of several elements, in some cases to a degree that is an order of magnitude higher than world averages. These include, arsenic, cadmium, chromium, copper, lead, mercury, uranium and zinc [5]. Several of these elements are of toxicological concern. The higher mass fractions of some of the potentially toxic elements are associated with bauxitic and terra rosa soils and intersect with the growing regions for several crops (see Fig. 1). Although this mineralization has occurred through natural surface processes [5], the implications for uptake by food crops are nonetheless of concern irrespective of origin.

The elemental content, including trace elements, of several Jamaican food crops, has been presented in a previous study [6]. The potential health risks associated with the consumption of these food stuffs was never fully investigated however. This study was undertaken to evaluate the risk from exposure to aluminium, arsenic, cadmium and lead through the consumption of Jamaican-grown foods, some of which are exported, using target hazard quotient (THQ) and hazard index (HI). Additionally, the target cancer risk (TCR) was also calculated for arsenic to determine the risk of cancer posed by the content of this element in these crops. The methodologies for THQ, HI and TCR have been used in several studies [7], [8], [9], [10] for various food types but sparingly if ever in foods from Latin America and the Caribbean.

## 2 Materials and methods

### 2.1 Sampling and preparation

Samples of ackee (*Blighia sapida*), banana (*Musa acuminate*), cabbage (*Brassica oleracea*), carrot (*Daucus carota*), cassava (*Manihot esculenta*), coco (*Xanthosoma sagittifolium*), dasheen (*Colocasia esculenta*), Irish potato (*Solanum tuberosum*), pumpkin (*Cucurbita pepo*), sweet pepper (*Capsicum annuum*), sweet potato (*Ipomoea batatas*), tomato (*Solanum lycopersicum*) and turnip (*Brassica rapa*) were collected from markets and farms island-wide. These samples were collected in labelled paper or plastic bags and transported to the food preparation laboratories at the International Centre for Environmental and Nuclear Sciences (ICENS). Samples were brushed to remove surface soil and any other potential sources of surface contamination, washed with tap water and carefully patted dry using clean paper towels. Peel and other non-edible portions were removed and the edible portion of each sample cut into smaller pieces. Samples were dried to constant weight at a temperature not exceeding 60 °C in an analytical laboratory oven and thereafter ground and homogenized using an automated agate mortar and pestle. Moisture content was determined using a subsample that was dried to constant weight. Ackee samples were treated in a similar manner as other samples with a notable exception. The edible portion of the ackee fruit is the fleshy extension of the seed referred to as the aril which was separated from the seed. This was analysed fresh.

### 2.2 Analysis

Samples were analysed by atomic absorption spectrophotometry (AAS) and instrumental neutron activation analysis (INAA).

#### 2.2.1 Atomic absorption spectropohotometry

Samples were prepared for analysis by Flame-AAS (Al), Graphite Furnace-AAS (Cd, Pb) and Hydride Generation-AAS (As) by acid digestion. 20 ml of 1:3 HCl:HNO3 was added to 1 g of sample in a 70 ml graduated polyethylene vial and allowed to stand overnight. The following day the samples were digested at 110 °C for 2 h using a ModBlock (CPI International) and made up to 50 ml. For ackee samples for the analysis of lead, 10 mL of HNO3 was added to 0.5 g of sample in an EasyPrep Teflon vial and allowed to stand for 1 h before digestion using a CEM MARS 5 microwave system (CEM Corporation, NC, USA). After cooling, samples were made up to 25 mL using deionized water. Acid digested samples were analysed using a PerkinElmer 5100PC Spectrophotometer (PerkinElmer, MA, USA) with Zeeman Background Correction. Calibration standards were prepared using Certiprep solutions (SPEX Certiprep, NJ, USA) in 2% HNO3. A matrix modifier was added to samples for the GFAAS analyses. The limits of detection (LODs) on a fresh weight basis ranged from 0.722–15.0 mg/kg for aluminium, 0.007–0.150 mg/kg for arsenic, 0.001–0.020 mg/kg for cadmium and 0.003–0.065 mg/kg for lead.

#### 2.2.2 Instrumental neutron activation analysis

Samples were analysed by INAA using the SLOWPOKE-2 nuclear reactor. For the determination of the short-lived radioisotope ^28^Al, approximately 0.5 g of sample was weighed out into pre-cleaned double polyethylene bags and heat sealed in pre-cleaned 7 cm^3^ polyethylene vials [11]. Each sample was irradiated for 3 min at a neutron flux of 5 × 10^11^ n cm^−2^s^−1^ and allowed decay periods of approximately 5 min before counting. For the longer-lived radioisotopes ^76^As and ^115^Cd approximately 1 g of sample was weighed out in pre-cleaned polyethylene capsules which were then heat sealed in 7 cm^3^ polyethylene vials and irradiated for 4 h at a neutron flux of 10 × 10^11^ n cm^−2^s^−1^ and allowed decay periods of 4 days. Samples were counted on hyper-pure germanium (HPGe) detectors with relative efficiencies ranging from 15% to 71%. The limits of detection (LODs) were 0.5 mg/kg for aluminium, 0.0005 for arsenic and 0.01 mg/kg for cadmium on a fresh weight basis. Lead was not analysed for by INAA.

### 2.3 Quality control

Approximately 10% of the samples were analysed in duplicate, with the differences between duplicates being less than 15%; at least one reagent blank was analysed in each batch in the case of AAS, and a certified reference material was also included in each analysis batch. Reference materials used for analysis of the elements were NIST (National Institute of Standards and Technology, MD, USA) 1573a − Tomato Leaves, NIST 1547–Peach Leaves and IAEA (International Atomic Energy Agency, Vienna, Austria) 336–Lichen. Recovery for reference materials used were within 10%.

### 2.4 Health risk assessment: estimated daily intake, target hazard quotient, hazard index and target cancer risk

#### 2.4.1 Estimated daily intake

The estimated daily intake (EDI) of the elements of interest (Al, As, Cd and Pb) were determined based on their average concentration in each food sample type and the daily intake in grams of the respective food items. Consumption data was estimated by accessing FAOSTAT (see Table 2). Food Balance/Food Supply-Crops primary equivalent data for Jamaica was retrieved for 2013, the year for which data was most recently compiled (see Table 2). For example, sweet potatoes were selected for the year 2013 which returned a food supply value of 12.96 kg/capita/year. This food supply value was divided by 365 (the number of days in the year) and the result multiplied by 1000 for conversion to grams. The result is an intake of 35.51 g/capita/day which is the food ingestion rate (F_IR_) of sweet potato in Jamaica. The following equation was used for EDI:

$$EDI=\frac{\left(C\times F_{IR}\right)}{BWa}$$

Where C is the fresh weight concentration of the element in the food type in mg/kg, *F_IR_* is the daily food ingestion rate in grams per day and *BWa* is the reference body weight of 70 kg.

#### 2.4.2 Target hazard quotient

The target hazard quotient (THQ) is defined as the ratio of exposure to the toxic element and the reference dose which is the highest level at which no adverse health effects are expected. The reference dose is specific to the trace element being assessed. The THQ describes the non-carcinogenic health risk posed by exposure to the respective toxic element. If the THQ is <1 then non-carcinogenic health effects are not expected. If, however, the THQ is >1 then there is a possibility that adverse health effects could be experienced. A THQ exceeding 1 is not a statistical probability that adverse non-carcinogenic health effects will occur. The THQ was estimated using the United States Environmental Protection Agency (US EPA) methodology based on the Region III risk-based concentration table.

$$THQ=\frac{E_{FR}\times Ed\times F_{IR}\times C}{RfD\times BWa\times ATn}\times 10^{-3}$$

Where E_FR_ is the exposure frequency to the trace element, Ed is the exposure duration (70 yrs), F_IR_ is the food ingestion rate in grams per day for the respective food item, C is the concentration in wet weight of the trace element in the given food item, R*f*D is the oral reference dose of the trace element in μg/g/day, BWa is the reference body weight of 70 kg and ATn is the averaged exposure time (365 days*70yrs) and 10^−3^ is the unit conversion factor (see Table 2).

#### 2.4.3 Hazard index

The hazard index (HI) is the sum of the individual target hazard quotients of the elements assessed for each food type. The HI assumes that the consumption of a particular food type would result in simultaneous exposure to several potentially toxic elements. Even if individual THQs for the elements in the food item are lower than unity individually the cumulative effect of consumption may result in adverse health effects. If the HI is >1 there is the potential for adverse non-carcinogenic health effects. The equation for HI is:

$$HI=\overset{i}{\underset{N=1}{\sum }}THQ_{n}$$

#### 2.4.4 Target cancer risk for arsenic

The target cancer risk (TCR) is used to assess the potential risk associated with exposure to carcinogenic agents throughout the lifetime exposure period. Instead of an oral reference dose, as is used for the determination of THQ, an oral slope factor is utilized. This factor determines, along with the dose of the carcinogen, the probability of excess cancer risk over the lifetime of the exposed individual. The equation for TCR is:

$$TCR=\frac{E_{FR}\times E_{D}\times F_{IR}\times C\times CPS_{O}}{BWa\times ATc}\times 10^{-3}$$

Where E_FR_ is the exposure frequency to arsenic, E_D_ is the exposure duration (70 yrs), *F_IR_* is the food ingestion rate in grams per day for the respective food item, C is the concentration in wet weight of the trace element in the given food item, *CPS_O_* is the oral cancer slope factor for inorganic arsenic of 1.5 (mg/kg)/day, BWa is the reference body weight of 70 kg, ATc is the averaged exposure time to the carcinogen (365 days*70yrs) and 10^−3^ is the unit conversion factor (see Table 2). The carcinogenicity of aluminium has not been established at this point [12] and so no oral cancer slope factor has been established. Currently no oral slope factor currently exists for cadmium and the US EPA has never established one for lead [13].

## 3 Results and discussion

### 3.1 Estimated daily intake, target hazard quotient, hazard index and global target hazard quotient

The aluminium, arsenic, cadmium and lead concentrations for the thirteen foodstuffs analysed are presented in Table 1. The aluminium content ranges from 2.58 mg/kg found in pumpkins to a high of 93.12 mg/kg in bananas. The arsenic content ranged from 0.001 mg/kg in cabbages to 0.104 mg/kg also in bananas. The cadmium content was analysed from 0.015 mg/kg in pumpkin samples to 0.286 mg/kg in turnip though both the mean cadmium content of tomatoes and ackee were close to this value at 0.266 and 0.248 mg/kg respectively. Cabbage samples had the lowest mean content of lead at 0.003 mg/kg with cassava samples having the highest mean content of lead at 0.100 mg/kg. All values are reported as fresh weight.

**Table 1:** The mean content of Al, As, Cd and Pb (mg/kg fresh weight) in selected Jamaican-grown food crops.

Food	Al	As	Cd	Pb
ackee	6.89	0.011	0.248	0.033
banana	93.12	0.104	0.057	0.010
cabbage	8.49	0.001	0.041	0.003
carrot	4.25	0.004	0.031	0.006
cassava	13.44	0.019	0.063	0.100
coco	3.28	0.006	0.079	0.017
dasheen	5.04	0.008	0.024	0.021
Irish potato	22.04	0.003	0.073	0.010
pumpkin	2.58	0.014	0.015	0.006
sweet pepper	7.27	0.002	0.157	0.005
sweet potato	34.23	0.006	0.096	0.054
tomato	12.89	0.012	0.266	0.021
turnip	36.69	0.007	0.286	0.006

**Table 2:** Parameters and variables used in the calculation of EDI, THQ and TCR.

Food	n	Efr (days)	ED (years)	F_IR_ (g/day)	BW (kg)	AT/c* (days)	Rfd (mg/kg day^−1^)				CPS_o_ (mg/kg day^−1^)
							Al	As	Cd	Pb	As
ackee	18	365	70	48.34	70	25550	1	0.0003	0.001	0.002	1.5
banana	15	365	70	34.71	70	25550	1	0.0003	0.001	0.002	1.5
cabbage	17	365	70	277.94	70	25550	1	0.0003	0.001	0.002	1.5
carrot	75	365	70	22.85	70	25550	1	0.0003	0.001	0.002	1.5
cassava	50	365	70	5.97	70	25550	1	0.0003	0.001	0.002	1.5
coco	45	365	70	213.56	70	25550	1	0.0003	0.001	0.002	1.5
dasheen	15	365	70	213.56	70	25550	1	0.0003	0.001	0.002	1.5
Irish potato	16	365	70	31.11	70	25550	1	0.0003	0.001	0.002	1.5
pumpkin	16	365	70	48.34	70	25550	1	0.0003	0.001	0.002	1.5
sweet pepper	12	365	70	48.34	70	25550	1	0.0003	0.001	0.002	1.5
sweet potato	69	365	70	35.51	70	25550	1	0.0003	0.001	0.002	1.5
tomato	129	365	70	30.66	70	25550	1	0.0003	0.001	0.002	1.5
turnip	11	365	70	22.85	70	25550	1	0.0003	0.001	0.002	1.5

* AT used for estimation of THQ, ATc used for estimation of TCR.

The calculations and results for the EDI (see Table 3) are based on a number of parameters; the uncertainty associated with the use of these variables have possibly resulted in an overestimation of the non-carcinogenic risk posed by the consumption of the elements assessed in this study. The EDI in this study is based on the food balance sheets of the most recent data available from the Food and

**Table 3:** Estimated Daily Intake of Al, As, Cd and Pb via the consumption of Jamaican-grown food crops.

Food	Estimated Daily Intake (μg/day/kg body weight)			
	Al (1)*	As (0.003)*	Cd (0.001)*	Pb (0.002)*
ackee	4.76	0.008	0.171	0.022
banana	46.17	0.051	0.028	0.005
cabbage	33.70	0.005	0.163	0.010
carrot	1.39	0.001	0.137	0.002
cassava	1.15	0.002	0.005	0.009
coco	10.00	0.019	0.240	0.051
dasheen	15.37	0.025	0.073	0.064
Irish potato	9.79	0.001	0.032	0.004
pumpkin	1.78	0.009	0.010	0.004
sweet pepper	5.02	0.001	0.109	0.003
sweet potato	17.37	0.003	0.049	0.027
tomato	5.65	0.005	0.116	0.009
turnip	11.98	0.002	0.093	0.002

* Oral reference dose in parentheses in mg/kg day^−1^.

Agriculture Organization [14]. Food balance sheets have inherent limitations. They may not consider food consumption by tourists, non-human consumption of food such as animal feed or industrial use of crops [15]. Food balance sheets assume uniform consumption without accounting for socioeconomic and cultural differences. There is also the assumption that availability equals consumption. This does not account for plate waste, spoilage, etc. which means the amount consumed is likely less than the number in the food balance sheet [16]. Finally, food balance sheets necessarily cannot consider all varieties of food. For example, in the case of FAOSTAT there is a category for bananas. Therefore, there is a food supply quantity for bananas specifically for Jamaica (12.67 kg/capita/year). However, for other categories there are more general terms such as starchy tubers (See Table 2). Several foods may fit this category resulting in an error in the estimate of consumption. These sources of error notwithstanding, the validity of food frequency questionnaires has long been questioned [17], [18] and so food balance sheets were selected as the more objective option.

The withdrawal of the provisional tolerable weekly intake (PTWI) for both inorganic arsenic and lead has left it necessary to find other methodologies to evaluate the risk associated with consumption of foods with significant levels of these and other potentially toxic elements. The calculation of the EDI and THQ are two such methodologies. To calculate the EDI and THQ an oral reference dose is necessary. As defined in the US EPA’s [19] *A Review of the Reference Dose and Reference Concentration Processes*, this is the “estimate (with uncertainty spanning perhaps an order of magnitude) of a daily oral exposure to the human population (including sensitive subgroups) that is likely to be without an appreciable risk of deleterious effects during a lifetime. It can be derived from the No Observed Adverse Effect Level (NOAEL), the Lowest Observed Adverse Effect Level (LOAEL), or benchmark dose, with uncertainty factors generally applied to reflect limitations of the data used” [19]. The oral reference doses used in this study were downloaded from the US EPA’s Regional Screening Level’s Generic Tables of May 2016 [20].

Aluminium has an oral reference dose of 1 mg/kg day^−1^. Food comprises greater than ninety percent (90%) of the non-occupational human exposure to aluminium [12]. Several studies have indicated neurotoxicity associated with long term exposure to aluminium although in many cases these are animal studies. The human studies tend to focus on the subset of patients undergoing dialysis and dialysis encephalopathy [21]. Although the association of aluminium with Alzheimer’s disease seems to be only a correlation, this element has been associated with brain aging and some neurodegenerative diseases such as Parkinson’s disease [21], [22]. The oral reference dose for inorganic arsenic is 0.0003 mg/kg day^−1^. As is the case with aluminium, the primary source of arsenic is through food for the non-occupationally exposed consumer [21]. Arsenic is more bioavailable in water than in food but can still be a significant contributor to the level of dietary exposure [23]. Seafood tends to have high levels of arsenic although the majority of this tends to be the less toxic organic species of arsenic. Larger percentages of inorganic arsenic have been found in market basket studies and the accumulation of inorganic arsenic in grains and produce may be very significant [24], [25], [26]. The oral reference dose for dietary cadmium is 0.001 mg/kg day^−1^. Cadmium is well known as a nephrotoxin with the incidence of renal tubular induced osteomalacia known as Itai Itai disease, a well-known example of the consequences of extreme dietary exposure to this metal [27]. Cadmium also has several effects on other systems of the human body [28]. The Integrated Risk Information System (IRIS) of the US EPA has the oral reference dose for lead and it compounds at 0.002 mg/kg day^−1^. Lead is a well-known neurotoxin that can accumulate in tissue including blood and bone causing a range of deleterious health effects [24].

**Fig. 2:**
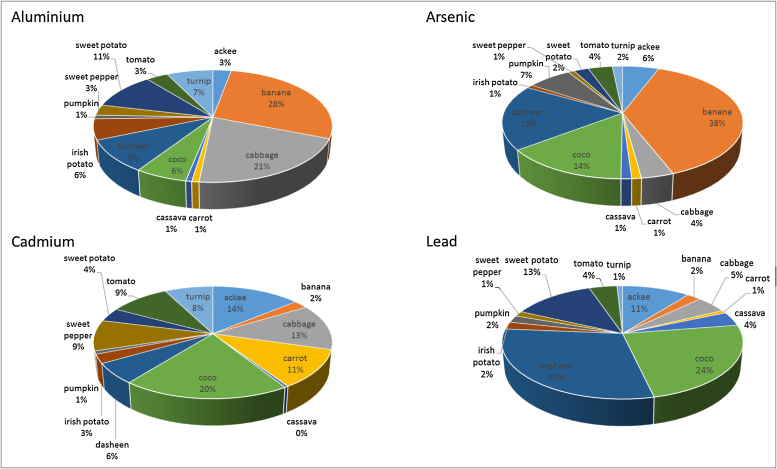
Percentage contribution of each foodstuff to the Target Hazard Quotient of Al, As, Cd and Pb respectively.

It is notable that no individual THQ for any of the foodstuffs analysed for any of the four elements is >1 (see Table 4). This indicates that in and of themselves consumption of the thirteen foods analysed presents no undue risk of non-carcinogenic health effects. For aluminium the THQ values range from 0.001 in cassava to 0.046 in bananas (Table 4). It is of interest that bananas exceed the aluminium content of other foodstuffs as shown in Table 1. In fact, bananas account for over 28% of the global hazard quotient of all foods for Al (Fig. 2). By comparison the root vegetables coco and dasheen only account for a cumulative 15% of the global target hazard quotient. Altogether, four crops account for 64% of the global target hazard quotient (see Fig. 2). Examining the individual contribution of each food crop to the global target hazard quotient for arsenic, indicates that banana contributes about 38%. With dasheen and coco contributing 19% and 14% respectively. Cumulatively these three crops contribute 71% of the global target hazard quotient for arsenic (see Fig. 2). In contrast to the previous elements cadmium shows a somewhat more even distribution. Coco accounts for 20% of the total THQ with ackees contributing 14%, cabbage at 13%, carrots at 11%, tomatoes at 9% and turnips at 8%. Still, these six food crops account for 75% of the GTHQ. The root crops coco and dasheen account for 54% of the GTHQ for lead with 24% and 30% respectively. With sweet potatoes at 13% and ackees at 11% these four crops contribute 78% of the GTHQ for lead.

**Table 4:** Target Hazard Quotient, Hazard Index and Global Target Hazard Quotient for Al, As, Cd and Pb analysed in Jamaican-grown food crops.

Food	Target Hazard Quotient (THQ)				Hazard Index (HI)
	Al	As	Cd	Pb	
ackee	0.005	0.025	0.171	0.011	0.213
banana	0.046	0.171	0.028	0.002	0.248
cabbage	0.034	0.017	0.163	0.005	0.218
carrot	0.001	0.005	0.137	0.001	0.144
cassava	0.001	0.005	0.005	0.004	0.016
coco	0.010	0.064	0.240	0.025	0.340
dasheen	0.015	0.084	0.073	0.032	0.204
Irish potato	0.010	0.004	0.032	0.002	0.049
pumpkin	0.002	0.031	0.010	0.002	0.045
sweet pepper	0.005	0.004	0.109	0.002	0.119
sweet potato	0.017	0.010	0.049	0.014	0.090
tomato	0.006	0.017	0.116	0.005	0.144
turnip	0.012	0.007	0.093	0.001	0.113
Global Target Hazard Quotient (GTHQ)	0.164	0.445	1.229	0.106	1.944

**Fig. 3:**
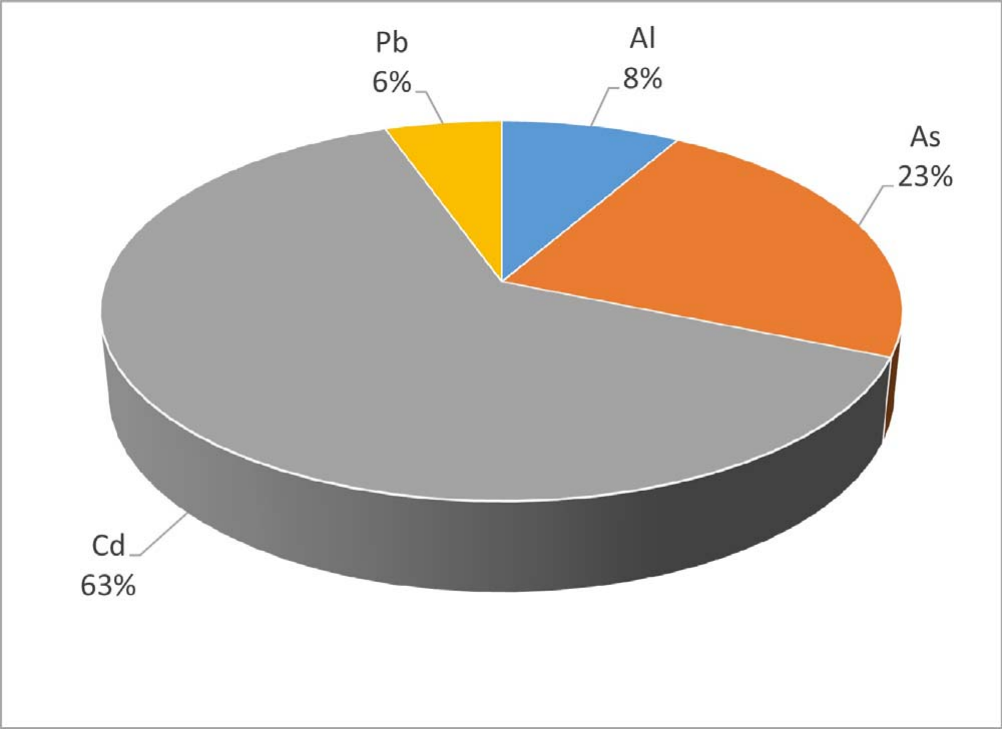
Average percentage contribution of Al, As, Cd and Pb to the Hazard Index.

The HI which considers the cumulative effect of the consumption of several potentially hazardous elements also does not exceed 1 (Table 4). The HI for the foods analysed for the four elements range from 0.016 for cassava to a high of 0.340 for coco (see Table 4). The estimated consumption patterns indicate cadmium contributing 63% to the HI of the foods analysed. This is followed by arsenic with 23% and then aluminium with 8% and lead with 6% (see Fig. 3). The EDI is evaluated against the oral reference dose or R*f*D. If one considers the average contribution of each food type to the respective R*f*Ds for each element analysed then this is not surprising. The average percentage contribution of the food types to the R*f*D for cadmium is almost 9.5%. By contrast the average percentage contribution by food crops to the arsenic R*f*d is 3.4%. The average aluminium percentage contribution is 1.3% and the foods analysed only contribute on average 0.8% to the R*f*D for lead. As per the definition of hazard quotient it is a ratio of the estimated exposure to a potentially hazardous substance to the level of that substance at which no adverse non-carcinogenic health effects are expected to occur. It is not a probability of risk or can it be converted into probability of risk or is it proportional to risk [29]. This is the same with HI. Because it is an aggregation of THQs it also cannot be looked at as probability of risk. Even if the THQs or HIs exceeded unity this would not equate to the certainty of adverse health effects. The HI approximates the cumulative risk of potential toxins. This should technically be looked at for substances with the same toxic mechanism and or target organ. While the route of exposure, oral, dietary exposure is the same, the mechanisms and major target organs are not the same. Sufficient evidence exists to add cadmium to the list of the other three elements as a neurotoxin [30], but the mechanisms are likely not similar.

It should be reiterated that the arsenic results presented in this study are total arsenic rather than inorganic arsenic, which was not determined. It is unlikely that the entire arsenic content of the various foods is inorganic and therefore the risk presented is also likely to be overstated. The THQ for arsenic is less than unity and it is likely that EDI, THQ and HI presented are overestimated as well.

The addition of several THQs can still add up to greater than the target level. This can happen because one food type for a particular toxic element exceeds unity or the addition of several THQs of sub-target level still sum to greater than unity. This would appear to be the case for the GTHQ of cadmium (see Table 4). Moreover, the combined or total HI is 1.944 (see Table 4) which is the same as the summation of the four GTHQs. According to the US EPA’s [31] *Human Health Risk Assessment Protocol for Hazardous Waste Combustion Facilities,* when this is the case an assessment is necessary to determine if the potential for non-carcinogenic health effects have been accurately evaluated. The cumulative exposure to multiple toxic elements may not necessarily be additive. With the additional potential for overestimation of consumption when summing multiple foods using food balance sheets, for example, assigning 213 grams per day for both coco and dasheen, each THQ may be very conservative resulting in an overestimated GTHQ and by extension total HI. By this metric of assessment however it is clear that cadmium is a major element for further consideration.

### 3.2 Target cancer risk

As previously stated the only element evaluated in this study for which the US EPA has established an oral cancer slope is arsenic. The TCR values range from 1.88E-06 in sweet peppers to 7.70E-05 in bananas (see Table 5). As is the case with the calculations for EDI, THQ and HI, the TCR for arsenic is based on the oral cancer slope for inorganic arsenic. Considering that the calculation was made using total arsenic it is likely that the TCR for inorganic arsenic is lower. Following on publications using 10^−6^ to 10^−4^ as the range for acceptable risk of developing cancer [32], [33], 10^−4^ was accepted as the upper limit for acceptable risk of developing cancer. No TCR result for any food type analysed exceeded 10^−4^. It is possible that if the TCR was based on inorganic arsenic content rather than total the TCR results would be lower. By summing the individual TCRs the result is 2.00 × 10^−4^. This cumulative TCR risk would exceed the 10^−4^ threshold and be cause for some concern. Terrestrial foods may have a wide range of inorganic arsenic content [26]. Chen et al. [34] assumed an inorganic content of 50% and following on this the cumulative TCR would be 10^−4^. With the possible overestimation in consumption data it is also likely that the cumulative TCR is actually under 10^−4^. The potential closeness to 10^−4^ means, however that further investigation may be warranted.

**Table 5:** Target Cancer Risk (TCR) for arsenic analysed in Jamaican-grown food crops.

Food	TCR
ackee	1.14E-05
banana	7.70E-05
cabbage	7.48E-06
carrot	2.19E-06
cassava	2.43E-06
coco	2.88E-05
dasheen	3.77E-05
Irish potato	1.95E-06
pumpkin	1.40E-05
sweet pepper	1.88E-06
sweet potato	4.69E-06
tomato	7.61E-06
turnip	3.19E-06

## 4 Conclusions

The food crops analysed presented no undue risk of adverse health effects, whether non-carcinogenic in the case of THQ for aluminium, arsenic, cadmium and lead or for cumulative health effects in terms of HI. The individual food crops analysed fall within the range of acceptable cancer risk for inorganic arsenic although the results are for total arsenic. It is possible that with the addition of targeted food surveys supplemental to the food balance sheets the estimated daily intake of these and other elements can be refined to more accurately represent the exposure of the Jamaican population. These targeted food studies could also be tailored to address special groups such as vegetarians and vegans whose consumption of these foods may be higher than the general public and for whom other foods will have to be considered. Future investigations are necessary to more accurately estimate the THQ and TCR for inorganic arsenic, to understand the risk of cadmium and its contribution to the total HI and to expand the food types for a comprehensive look at the safety of Jamaican food.
